# Electrochemistry of Graphene Nanoplatelets Printed Electrodes for Cortical Direct Current Stimulation

**DOI:** 10.3389/fnins.2020.594235

**Published:** 2020-10-29

**Authors:** Andrzej Pepłowski, Sanchit Rathi, Bartosz Piotrkowski, Robert Ziółkowski, Daniel Janczak, Jakub Krzemiński, Michael Brosch, Małgorzata Jakubowska

**Affiliations:** ^1^Institute of Metrology and Biomedical Engineering, Faculty of Mechatronics, Warsaw University of Technology, Warsaw, Poland; ^2^Research Group Comparative Neuroscience, Leibniz Institute for Neurobiology, Magdeburg, Germany; ^3^Faculty of Electrical Engineering and Information Technology, Otto von Guericke University, Magdeburg, Germany; ^4^Chair of Medical Biotechnology, Faculty of Chemistry, Warsaw University of Technology, Warsaw, Poland; ^5^Centre for Advanced Materials and Technologies, Warsaw University of Technology, Warsaw, Poland; ^6^Center for Behavioral Brain Sciences, Otto von Guericke University, Magdeburg, Germany

**Keywords:** flexible electrodes, graphene nanoplatelets, neural stimulation, voltammetry, screen-printing

## Abstract

Possible risks stemming from the employment of novel, micrometer-thin printed electrodes for direct current neural stimulation are discussed. To assess those risks, electrochemical methods are used, including cyclic voltammetry, square-wave voltammetry, and electrochemical impedance spectroscopy. Experiments were conducted in non-deoxidized phosphate-buffered saline to better emulate living organism conditions. Since preliminary results obtained have shown unexpected oxidation peaks in 0–0.4 V potential range, the source of those was further investigated. Hypothesized redox activity of printing paste components was disproven, supporting further development of proposed fabrication technology of stimulating electrodes. Finally, partial permeability and resulting electrochemical activity of underlying silver-based printed layers of the device were pointed as the source of potential tissue irritation or damage. Employing this information, electrodes with corrected design were investigated, yielding no undesired redox processes.

## Introduction

For both therapeutic reasons and fundamental neuroscientific study, the capacity to alter brain states is essential. With electrodes that are invasively inserted into the brain or placed outside the skull, electrical stimulation is accomplished. The electrodes produce electrical fields that reach the brain where they influence neuronal electrical activity and change neuronal electrical communication. Most electrical stimulation protocols employ alternating currents (ACs) that evoke action potentials in neurons. They travel along the axons and affect both nearby and remote neurons. An alternative type of electrical stimulation is direct current (DC) stimulation (Creutzfeldt et al., 1962; Rosen and Stamm, 1972), which has become of interest to one of the authors (Oshurkova et al., 2008; Brosch et al., 2011) because it changes the polarization of cortical neurons underneath the working electrode. In contrast to AC stimulation, less is known on the physiological and behavioral consequences of DC stimulation. This is partly due to a lack of availability of appropriate electrodes for this type of stimulation.

The goal of the present research is to develop and fabricate intracortical DC stimulation electrodes using sophisticated material combination and evaluate their electrochemical characteristic. This had to be done because initially, we used traditional silver/silver chloride pellet electrodes for cortical DC stimulation and observed that the electrolysis reduced their effective life span and usability. In addition, the electrode’s surface corrosion stemming from the electrolysis presented not only as disadvantageous due to the reduced lifetime but also as a potential risk of inducing inflammatory reaction in the nervous system by released silver ions (Wu and Tang, 2018). This can be mitigated by using higher charge injection capacity of DC stimulation electrodes that would allow more charge to be delivered to the surroundings without irreversible reactions. We also observed that the growth of a thick scar tissue above the dura mater over weeks increased the distance between the electrode’s surface and the targeted brain region, and thus a higher amplitude current was required to produce the same effect on the brain. Thus, a need to place the electrode into the subdural space arose, as it would allow the employment of lower amplitudes thanks to reduced distance to the area being stimulated. However, getting the electrode to the desired place requires surgical implantation and consequently the use of very thin electrodes. To the best of our knowledge, such electrodes are currently not commercially available but have to be custom built to our specifications. Such devices, besides their dimensions, should exhibit appropriate flexibility to conform to the brain’s topography (Ludwig et al., 2006; Apollo et al., 2015; Castagnola et al., 2015; Kassegne et al., 2015). To achieve this, printed electronics technology presents one of the potential solutions (Adly et al., 2018). This approach also allows the employment of superior materials, such as carbon (Vomero et al., 2017), for the fabrication of electrodes. In contrast to metallic electrodes, carbon allotropes are safer in terms of potential inflammatory effects, as well as exhibiting higher charge injection capacity than, e.g., Pt electrodes (Vomero et al., 2016). Among those are the many graphene materials, which are being investigated for their potential contribution toward low electrode impedance (Park et al., 2014; Apollo et al., 2015). In spite of these advantages, every printed composite should be examined to determine its safety and usability as the properties of electrode–tissue interface may vary significantly with the electrode’s surface structure, material, etc. (Harris et al., 2018) and thus may cause various types of tissue damage (Kumsa et al., 2019). For example, molecular oxygen that evolved due to oxidation of water (Kumsa et al., 2016) may in turn lead to oxidation of tissue. Other mechanisms may lead to electrochemical production of toxic species (Fridman and Santina, 2013) or forced activation of cells that cannot withstand high levels of stimulation (Günter et al., 2019). Finally, electrode corrosion may result in electrode material particles being released in the tissue environment (Wissel et al., 2018), leading to inflammatory reaction.

Electrochemistry for examination of neural stimulation electrodes is a long-established standard procedure (Brummer and Turner, 1977; Cogan, 2008; Kassegne et al., 2015; Harris and Wallace, 2019). Reactions involving oxygen evolution, electrode decomposition, or other unintended redox processes can be easily observed in cyclic voltammetry (CV) or square-wave voltammetry (SWV) plots, yielding characteristics of the electrode in desired potential window. In addition, electrochemical impedance spectroscopy (EIS) can reveal information about changes in the electrode’s surface accessibility, and from that, processes taking place there could be concluded (Lasia, 2002). Although for a long time metallic electrodes were investigated in H_2_SO_4_, Hudak et al. (2010) demonstrated that such experiments could as well be conducted in phosphate-buffered saline (PBS), which additionally better emulates conditions found within an organism than H_2_SO_4_. Mainly, it reflects the natural ability of a living system to maintain a certain pH level—a factor that affects significantly electrochemical results (Skoog et al., 2013).

In this work, we have applied electrochemical test methods (mentioned in detail in the subsequent sections) to evaluate the properties of flexible, printed graphene nanoplatelet (GNP) electrodes. Our primary goal was to determine if there are any shortcomings, such as redox processes or material decomposition, that may hinder the use of the developed electrodes in performing intracortical DC stimulation. The study also looked for ways to find solutions if such gaps were to be found.

## Methods

### Reagents and Materials

Phosphate-buffered saline was prepared using sodium chloride (NaCl), sodium phosphate monobasic monohydrate (H_2_NaPO_4_⋅H_2_O), and sodium phosphate dibasic heptahydrate (HNa_2_PO_4_⋅7H_2_O) (all from Sigma-Aldrich). Ingredients were dissolved in distilled water in proportion 0.13 M NaCl, 0.022 M H_2_NaPO_4_⋅H_2_O, and 0.081 M HNa_2_PO_4_⋅7H_2_O, yielding PBS with pH = 7.2.

Potassium hexacyanoferrate(II) trihydrate (K_4_[Fe(CN)_6_]⋅ 3H_2_O) and potassium hexacyanoferrate (III) (K_3_[Fe(CN)_6_]) (both from Fluka)^TM^ were used to prepare the second buffer (K_3_[Fe(CN)_6_]/K_4_[Fe(CN)_6_]), both in 25 mM concentration.

Screen-printing pastes for conductive layer of neurostimulation electrodes were composed of polymethyl methacrylate (PMMA; BASF GmbH) polymer matrix dissolved in butyl diglycol acetate (OKB, Sigma-Aldrich)^®^ as a vehicle with silver micro-flakes as conducting filler. Silver printing paste was purchased from Novelinks (Poland).

### Preparation of Printing Paste

Carbon-based screen-printing pastes for active layer of electrodes were likewise prepared using dissolved PMMA granulate (8 wt.%) in the OKB. GNPs with mean diameter 15 μm and thickness below 10 nm (XG Sciences, Inc., United States) were used to prepare the 12.5 wt.% GNP paste by adding them into the vehicle solution of PMMA. Prior to addition, GNPs were sonicated in acetone for 20 min. After 10 min of sonication, dispersing agent (DA) was added to one part of the suspension in an amount of 2 wt.% of GNP mass. As a DA, AKM-0531 (NOF, Corp., Japan) was used. After sonication, GNP suspension was dried at 90°C to evaporate acetone. DA was used to further enhance deagglomeration, chiefly for two reasons: agglomerated GNPs lead to poorer printability (Dybowska-Sarapuk et al., 2015) of the final paste and layers printed with such paste exhibit much lower electrical conductivity (Dybowska-Sarapuk et al., 2018). Dry GNPs were then added to PMMA solution and hand-mixed in agate mortar for 10 min. Finally, paste was rolled in the three-roll-mill with silicon carbide (*SiC*) rolls and 5 μm gap between rolls to obtain homogenous compositions and exclude the possibility of agglomeration of the GNPs. Thus, GNP and GNP/DA pastes were obtained used for fabrication of screen-printed SP-GNP and SP-GNP/DA electrodes.

### Fabrication of the Electrodes

For all printing steps, 77 T polyester screens were used, fabricated by Maroka (Poland).

SP-GNP and SP-GNP/DA electrodes were fabricated on 25.4 μm thick polyimide foil Kapton^®^ 100HN (DuPont Poland). Firstly, the holes were laser-drilled in the foil for further printing of vias between electric contacts and GNP electrodes. Secondly, on one side of the foil, silver paths were printed, and then on the other side, using the same method of screen printing, GNP electrodes were deposited using GNP and GNP/DA pastes. Finally, the side with silver paths was isolated with heat-curable 8155 paste (DuPont Poland).

To assess the hypothesis stated during the described experiments (Dispersing Agent Electrochemistry and Silver Contacts Electrochemistry sections), another version of SP-GNP/DA^∗^ electrodes was also prepared, with GNP/DA paste employed as well to print contacts between electrode layers and conducting routes. Thus, the silver layer was absent beneath the part designed to contact tissue directly.

The GCE/GNP and GCE/GNP/DA working electrodes were fabricated on a glassy-carbon electrode (GCE) by firstly polishing and rinsing GCE in distilled water, and in the next step, it was rinsed in ultrasound. Next, sonicated GNP and GNP/DA suspensions in acetone were drop-casted (2 μl) on the GCE surface and dried at 40°C for 30 min.

### Measurements

Methods employed to investigate electrochemical properties of fabricated electrodes were CV and SWV performed in both types of buffer (K_3_[Fe(CN)_6_]/K_4_[Fe(CN)_6_] and dimethyl sulfoxide [DMSO]). EIS of SP-GNP and SP-GNP/DA electrodes was also performed in K_3_[Fe(CN)_6_]/K_4_[Fe(CN)_6_]. In every electrochemical experiment described, Ag/AgCl(s) 1 M KCl reference electrode was used.

Before every series of measurements, a previously unused electrode was prepared by washing with distilled water and drying at 120°C for 15 min. Gold auxiliary electrode was heated in fire and then washed in acetone, followed by rinsing with water.

## Results and Discussion

### Assessment of the Influence of Dissolved O_2_ and Potential Range on the Electrodes’ Performance

**FIGURE 1:**
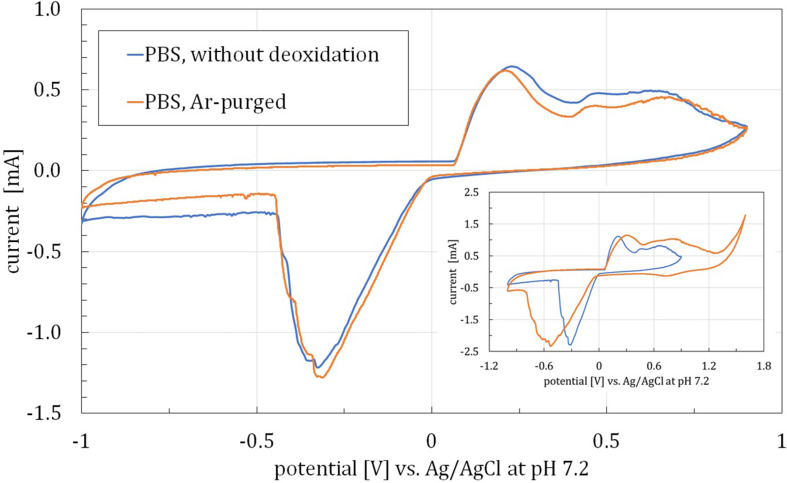
CV curves obtained for SP-GNP/DA electrodes with and without argon-purging. Inset: CV curves obtained for two potential ranges. Both experiments conducted in PBS with 0.13M NaCl, pH = 7.2.

Since in the living tissue the oxygen level can vary, the electrodes’ performance was examined in two PBS samples. One of those was kept in an open vessel during measurements to allow oxygen dissolution in the PBS. The second sample was subjected to an argon purging, by diffusing Ar in the solution for 30 min and then keeping the gas flow over the surface of the sample throughout the measurements. As it is visible on the overlaid CV plots (Figure 1), there are few differences between the electrode processes in both high and low O_2_ concentrations in the sample. This may be explained by the lack of redox activity of PBS components in the applied potential window (−1 to 0.9 V). Thus, a wider window (−1 to 1.6 V) was also used, as shown on the inset in Figure 1. For the potentials above 1.3 V, current corresponding to O_2_ evolution was observed. Thus, it may be concluded that 1.3 V is the upper limit for applied potential when stimulating tissue with fabricated electrodes for aqueous solutions. In both cases, wide oxidation regions are present above 0.4 V, which may be interpreted as oxidation of functional groups on GNP surface (Lee et al., 2017) or oxidation of Ag, which is contained in the bottom layer of SP-GNP/DA electrodes. The cathodic peak, observed below 0 V, may correspond to three overlapping processes: oxidized functional groups reduction, hydrogen adsorption, and H_2_ evolution. Besides that, one anodic peak was observed in 0–0.4 V range, which was subjected to further examination as described further. Finally, since neither deoxidation nor wider potential window altered the obtained results significantly, in the following experiments, −1 to 0.9 V range and PBS solution without gas-purging were used.

### Investigation of 0–0.4 V Oxidation Peaks

**FIGURE 2:**
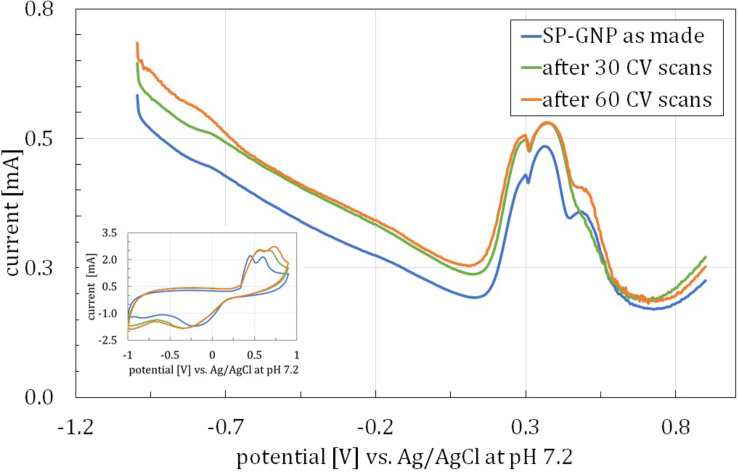
SWV curves for a single SP-GNP/DA before any CV measurements and after 30 and 60 CV scans, square-wave frequency = 15 Hz, amplitude = 25 mV. Inset: CV curves for analogically treated SP-GNP/DA electrode. Both experiments conducted in PBS with 0.13M NaCl, pH = 7.2.

**FIGURE 3:**
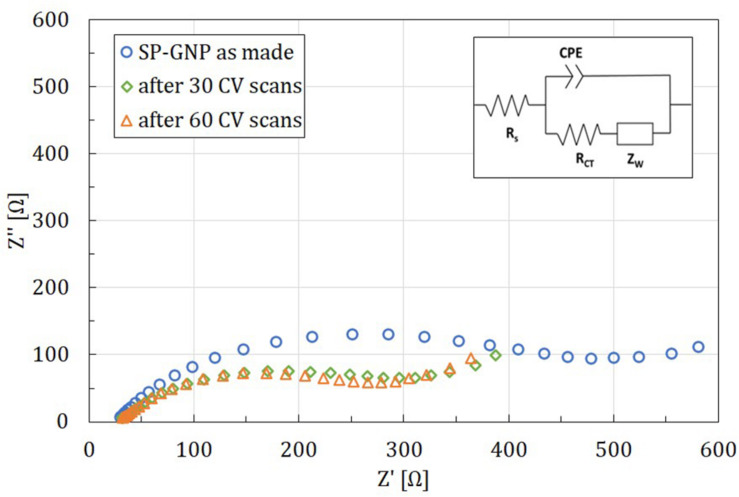
Electrochemical impedance spectroscopy results for a single SP-GNP/DA electrode before any CV measurements and after 30 and 60 CV scans in K_3_[Fe(CN)_6_]/K_4_[Fe(CN)_6_], low frequency = 1 Hz, high frequency = 100 kHz.

To determine the cause of 0–0.4 V oxidation peak, SWV measurements were performed, revealing the multi-process origin of the peak. To investigate the possible occurrence of intermediate processes, between SWV measurements, a series of 30 CV scans was carried out. Results of the former are shown on the main plot in Figure 2 and of the latter on the inset, presenting the last CV plot in each series. After each CV series, EIS (Figure 3) was also performed to investigate electrode surface accessibility, indicated by charge transfer resistance, R_*CT*_. It was then observed that between the first and second SWV measurements, i.e., after 30 CV scans, the most pronounced change was observed, whereas after another 30 CV scans, an alleged three-step redox process reached equilibrium to some measure. In that case, two sub-peaks present in the lower potential region of the peak were almost identical, with only the third one increasing its current. In addition, a significant decrease in R_*CT*_ was observed corresponding to the first scanning series (ΔR_*CT*_ = 183.5 Ω), whereas it only slightly decreased after the second one (ΔR_*CT*_ = 17.3 Ω). It was then presumed that the compound(s) undergoing the observed redox processes might be covering the electrode’s surface with its second and third forms hindering the access to the electrode to a lesser extent. Nevertheless, such alleged species presence on the electrode for direct neural stimulation was recognized as a potential risk and so demanded explanation and elimination.

### Dispersing Agent Electrochemistry

It was hypothesized that the species undergoing the redox process in 0–0.4 V range was the DA employed for printing paste. Since it helps deagglomerate GNPs by coulombic forces, its oxidation might have caused the observed impedance decrease. To further investigate that, GCE/GNP and GCE/GNP-DA were used for CV measurements in −1 to 0.9 V range in PBS. Since in the obtained results no significant oxidation peaks were observed that could be linked to DA reactions (data not shown), it was then concluded that this compound was not the source of 0–0.4 V peaks in previous measurements. From this, it may also be inferred that DA employed in the fabrication of electrodes would not undergo any potentially dangerous process during neural stimulation.

### Silver Contacts Electrochemistry

**FIGURE 4:**
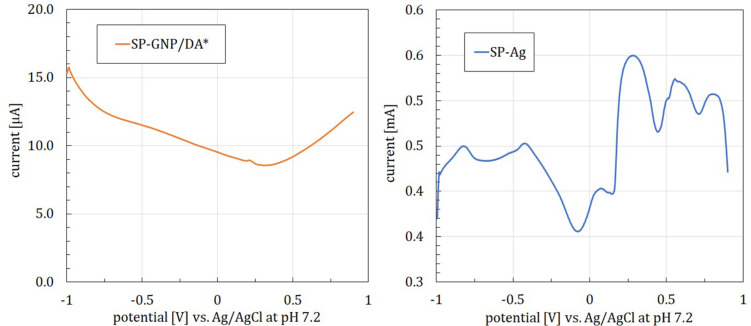
SWV curves for a screen-printed graphene electrode without silver conducting routes directly beneath the electrode layer (SP-GNP/DA*, left) and a screen-printed silver electrode (SP-Ag, right), registered in PBS with 0.13M NaCl, pH = 7.2, square-wave frequency = 15 Hz, amplitude = 25 mV.

Since the hypothesis of DA being the source of 0–0.4 V oxidation peaks was rejected, another presumption was made, namely, that the GNP printed layer is not entirely impermeable. Thus, silver-containing conductive routes lying beneath the GNP layer would be exposed to the solution. To assess this hypothesis, SP-GNP/DA^∗^ was employed. SWV measurements conducted with those yielded no oxidation peaks (Figure 4, left). Analogical measurements with SP electrode with no GNP layer and thus having the Ag layer directly exposed to the solution yielded pronounced redox activity peaks (Figure 4, right). Those were more distinctly separable than the ones observed previously (Figure 2), which can be explained by the SP-Ag electrode’s surface being more easily accessible. Further supporting the hypothesis of Ag causing 0–0.4 V peaks are the findings by Choi and Luo (2011), describing complicated redox reactions occurring on the bulk silver electrode within this potential range, which involve Ag oxide as well as chloride formation.

## Conclusion

The presence of silver directly beneath the electrode layer can lead to redox reactions and thus presents a risk to a living tissue. It was concluded that the main cause of it is the partial permeability of the printed layers. This risk can be eliminated by proper stimulating electrode design, i.e., extending to overlap of insulating layer over electrical contacts. On the other hand, graphene nanoplatelets are confirmed as a safe material for preparing printing pastes to be employed in electrodes for direct neural stimulation. Moreover, DA used with GNPs was also confirmed to undergo no electrochemical processes in the examined −1 to 0.9 V potential range. It is also a valuable information, as such addition enhances printed electrode conductivity and facilitates fabrication process. The presented flexible GNP-based electrode can be deemed as electrochemically safe for direct neural stimulation while meeting requirements of flexibility and thickness, needed for implanting in the subdural space. This tool may thus prove advantageous for further development of DC brain stimulation.

## Data Availability Statement

The original contributions presented in the study are included in the article/supplementary material, further inquiries can be directed to the corresponding author.

## Author Contributions

AP and BP contributed by conducting measurements and writing the manuscript. SR, DJ, and JK designed the two-layer printed electrode structure. DJ and JK contributed by fabrication of all electrodes. RZ contributed in designing electrochemical measurements and discussing results. MB and MJ supervised the research and reviewed the article in the neurobiological and technological aspects, respectively. All authors contributed to the article and approved the submitted version.

## Conflict of Interest

The authors declare that the research was conducted in the absence of any commercial or financial relationships that could be construed as a potential conflict of interest.

## References

[B1] Adly N. Weidlich S. Seyock S. Brings F. Yakushenko A. Offenhäusser A. (2018). Printed microelectrode arrays on soft materials: from PDMS to hydrogels. *Npj Flex. Electron.* 2:15 10.1038/s41528-018-0027-z

[B2] Apollo N. V. Maturana M. I. Tong W. Nayagam D. A. X. Shivdasani M. N. Foroughi J. (2015). Soft, flexible freestanding neural stimulation and recording electrodes fabricated from reduced graphene oxide. *Adv. Funct. Mater.* 25 3551–3559. 10.1002/adfm.201500110

[B3] Brosch M. Selezneva E. Scheich H. (2011). Formation of associations in auditory cortex by slow changes of tonic firing. *Hear. Res.* 271 66–73. 10.1016/J.HEARES.2010.05.003 20488230

[B4] Brummer S. B. Turner M. J. (1977). Electrochemical considerations for safe electrical stimulation of the nervous system with platinum electrodes. *IEEE Trans. Biomed. Eng. BME* 24 59–63. 10.1109/TBME.1977.326218 851475

[B5] Castagnola E. Maiolo L. Maggiolini E. Minotti A. Marrani M. Maita F. (2015). PEDOT-CNT-coated low-impedance, ultra-flexible, and brain-conformable micro-ECoG arrays. *IEEE Trans. Neural Syst. Rehabil. Eng.* 23 342–350. 10.1109/TNSRE.2014.2342880 25073174

[B6] Choi Y.-J. Luo T.-J. M. (2011). Electrochemical properties of silver nanoparticle doped aminosilica nanocomposite. *Int. J. Electrochem.* 2011 1–6. 10.4061/2011/404937

[B7] Cogan S. F. (2008). Neural stimulation and recording electrodes. *Annu. Rev. Biomed. Eng.* 10 275–309. 10.1146/annurev.bioeng.10.061807.160518 18429704

[B8] Creutzfeldt O. D. Fromm G. H. Kapp H. (1962). Influence of transcortical d-c currents on cortical neuronal activity. *Exp. Neurol.* 5 436–452. 10.1016/0014-4886(62)90056-0 13882165

[B9] Dybowska-Sarapuk L. Kielbasinski K. Arazna A. Futera K. Skalski A. Janczak D. (2018). Efficient inkjet printing of graphene-based elements: influence of dispersing agent on ink viscosity. *Nanomaterials* 8:602. 10.3390/nano8080602 30096800 PMC6116204

[B10] Dybowska-Sarapuk Ĺ Janczak D. Wróblewski G. Słoma M. Jakubowska M. (2015). “The influence of graphene screen printing paste’s composition on its viscosity,” in *XXXVI Symposium on Photonics Applications in Astronomy, Communications, Industry, and High-Energy Physics Experiments 2015 9662 (September 2015): 966242*, Wilga, 10.1117/12.2204739

[B11] Fridman G. Y. Santina C. C. D. (2013). Safe direct current stimulation to expand capabilities of neural prostheses. *IEEE Trans. Neural Syst. Rehabil. Eng.* 21 319–328. 10.1109/TNSRE.2013.2245423 23476007 PMC4050981

[B12] Günter C. Delbeke J. Ortiz-Catalan M. (2019). Safety of long-term electrical peripheral nerve stimulation: review of the state of the art. *J. NeuroEng. Rehabil.* 16:13. 10.1186/s12984-018-0474-8 30658656 PMC6339286

[B13] Harris A. R. Newbold C. Carter P. Cowan R. Wallace G. G. (2018). Measuring the effective area and charge density of platinum electrodes for bionic devices. *J. Neural Eng.* 15 1–12. 10.1088/1741-2552/aaba8b 29595147

[B14] Harris A. R. Wallace G. G. (2019). Electrochemical methods for analysing and controlling charge transfer at the electrode–tissue interface. *Curr. Opin. Electrochem.* 16 143–148. 10.1016/j.coelec.2019.07.001

[B15] Hudak E. M. Mortimer J. T. Martin H. B. (2010). Platinum for neural stimulation: voltammetry considerations. *J. Neural Eng.* 7:26005 10.1088/1741-2560/7/2/026005 20208126

[B16] Kassegne S. Vomero M. Gavuglio R. Hirabayashi M. Özyilmaz E. Nguyen S. (2015). Electrical impedance, electrochemistry, mechanical stiffness, and hardness tunability in glassy carbon MEMS M ECoG electrodes. *Microelectron. Eng.* 133 36–44. 10.1016/J.MEE.2014.11.013

[B17] Kumsa D. W. Bhadra N. Hudak E. M. Kelley S. C. Untereker D. F. Mortimer J. T. (2016). Electron transfer processes occurring on platinum neural stimulating electrodes: a tutorial on the i(Ve) profile. *J. Neural Eng.* 13:052001 10.1088/1741-2560/13/5/052001 27518125

[B18] Kumsa D. W. Hudak E. M. Bhadra N. Mortimer J. T. (2019). Electron transfer processes occurring on platinum neural stimulating electrodes: pulsing experiments for cathodic-first, charge-imbalanced, biphasic pulses for 0.566≤k ≤2.3 in rat subcutaneous tissues. *J. Neural Eng.* 16:026018. 10.1088/1741-2552/aaf931 30560809

[B19] Lasia A. (2002). “Electrochemical impedance spectroscopy and its applications,” in *Modern Aspects of Electrochemistry*, eds Conway B. E. Bockris J. O. White R. E. (Boston, MA: Kluwer Academic Publishers), 143–248. 10.1007/0-306-46916-2_2

[B20] Lee C.-S. Yu S. H. Kim T. H. (2017). One-step electrochemical fabrication of reduced graphene oxide/gold nanoparticles nanocomposite-modified electrode for simultaneous detection of dopamine, ascorbic acid, and uric acid. *Nanomaterials* 8:17. 10.3390/nano8010017 29301209 PMC5791104

[B21] Ludwig K. A. Uram J. D. Yang J. Martin D. C. Kipke D. R. (2006). Chronic neural recordings using silicon microelectrode arrays electrochemically deposited with a poly(3,4-Ethylenedioxythiophene) (PEDOT) film. *J. Neural Eng.* 3 59–70. 10.1088/1741-2560/3/1/007 16510943

[B22] Oshurkova E. Scheich H. Brosch M. (2008). Click train encoding in primary and non-primary auditory cortex of anesthetized macaque monkeys. *Neuroscience* 153 1289–1299. 10.1016/j.neuroscience.2008.03.030 18423884

[B23] Park D.-W. Schendel A. A. Mikael S. Brodnick S. K. Richner T. J. Ness J. P. (2014). Graphene-based carbon-layered electrode array technology for neural imaging and optogenetic applications. *Nat. Commun.* 5:5258. 10.1038/ncomms6258 25327513 PMC4218963

[B24] Rosen S. C. Stamm J. S. (1972). Transcortical polarization: facilitation of delayed response performance by monkeys. *Exp. Neurol.* 35 282–289. 10.1016/0014-4886(72)90154-9 4624141

[B25] Skoog D. A. West D. M. Holler F. J. Crouch S. R. (2013). *Fundamentals of Analytical Chemistry.* Available online at: http://repository.fue.edu.eg/xmlui/bitstream/handle/123456789/2829/9085.pdf?sequence=1&isAllowed=y (accessed September 26, 2019).

[B26] Vomero M. Castagnola E. Ciarpella F. Maggiolini E. Goshi N. Zucchini E. (2017). Highly stable glassy carbon interfaces for long-term neural stimulation and low-noise recording of brain activity. *Sci. Rep.* 7:40332. 10.1038/srep40332 28084398 PMC5234039

[B27] Vomero M. Castagnola E. Maggiolini E. Ciarpella F. Rembado I. Goshi N. (2016). A direct comparison of glassy carbon and PEDOT-PSS electrodes for high charge injection and low impedance neural interfaces. *Adv. Sci. Technol.* 102 68–76. 10.4028/www.scientific.net/AST.102.68

[B28] Wissel K. Brandes G. Pütz N. Angrisani G. L. Thieleke J. Lenarz T. (2018). Platinum corrosion products from electrode contacts of human cochlear implants induce cell death in cell culture models. *PLoS One* 13:e0196649. 10.1371/journal.pone.0196649 29763442 PMC5953457

[B29] Wu T. Tang M. (2018). The inflammatory response to silver and titanium dioxide nanoparticles in the central nervous system. *Nanomedicine* 13 233–249. 10.2217/nnm-2017-0270 29199887

